# Core-shell magnetoelectric nanorobot – A remotely controlled probe for targeted cell manipulation

**DOI:** 10.1038/s41598-018-20191-w

**Published:** 2018-01-29

**Authors:** Soutik Betal, Amit Kumar Saha, Eduardo Ortega, Moumita Dutta, Anand Kumar Ramasubramanian, Amar Singh Bhalla, Ruyan Guo

**Affiliations:** 1 Department of Electrical and Computer Engineering, University of Texas at San Antonio, San Antonio, TX 78249 USA; 2 Department of Biomedical Engineering, University of Texas at San Antonio, San Antonio, TX 78249 USA; 3 Department of Biomedical, Chemical, and Materials Engineering, San José State University, San José, CA 95192 USA; 4 Department of Physics and Astronomy, University of Texas at San Antonio, San Antonio, TX 78249 USA

## Abstract

We have developed a remotely controlled dynamic process of manipulating targeted biological live cells using fabricated core-shell nanocomposites, which comprises of single crystalline ferromagnetic cores (CoFe_2_O_4_) coated with crystalline ferroelectric thin film shells (BaTiO_3_). We demonstrate them as a unique family of inorganic magnetoelectric nanorobots (MENRs), controlled remotely by applied a.c. or d.c. magnetic fields, to perform cell targeting, permeation, and transport. Under a.c. magnetic field excitation (50 Oe, 60 Hz), the MENR acts as a localized electric periodic pulse generator and can permeate a series of misaligned cells, while aligning them to an equipotential mono-array by inducing inter-cellular signaling. Under a.c. magnetic field (40 Oe, 30 Hz) excitation, MENRs can be dynamically driven to a targeted cell, avoiding untargeted cells in the path, irrespective of cell density. D.C. magnetic field (−50 Oe) excitation causes the MENRs to act as thrust generator and exerts motion in a group of cells.

## Introduction

Targeted single cell electroporation and cell therapy are two revolutionary techniques in the field of medicinal science. Electroporation, or electro-permeabilization, is a microbiological technique in which an electrical field is introduced to affect the permeability of the cell membrane, allowing chemicals, drugs, or DNA to be introduced into the cell^1,2^. This is primarily due to reversible and irreversible nanoscale defect or nanopore formation on cell membranes^3^. It had been shown that irreversible electroporation can be used for minimally invasive treatment of aggressive cutaneous tumours implanted in mice^4^ or for the transportation of small molecule drugs, proteins or siRNAs and antisense oligonucleotides. Exosomes electroporated to contain such cargo can cross the blood brain barrier, thus addressing the issue of poor delivery of medications to the central nervous system to treat Alzheimer’s, Parkinson’s disease and brain cancer among other diseases^5,6^.

Advances in our understanding of the biophysical molecular mechanisms behind major diseases have led to the development of a wide array of cell-based therapies to deliver a therapeutic agent such as a modified, repopulating stem cell or a protein or virus^7^. Moreover, exogenous stem cell-based therapies hold potential to revolutionize medicine by restoring tissue and organ function^8,9^. Cell-based therapy could be used to help prevent the human body from rejecting transplanted organs, curing Parkinson’s disease and cancer treatment^10–13^. If the site of transplantation to be regenerated, however is not confined to one area in the body or is in a tissue inaccessible by direct injection of cells, such cell-based therapies must be administered systemically^14^. Previous studies have shown that magnetic nanoparticles (MNPs) can be injected systemically and attracted to a target tissue in mice by the application of remote magnetic field^15^. Researchers have previously showed that super paramagnetic iron oxide nanoparticle (SPION)-loaded human macrophages could be attracted from the circulation into tumours in mice using such an approach^16^. Techniques have also been proposed for vascular repair by circumferential cell therapy using MNPs and tailored magnets^17^. Some other applications of MNPs include using Zn_0.4_Fe_2.6_O_4_ cubic magnetic nanoparticles (c-MNPs) for remote mechanical control of the position of the stereocilia of an inner ear hair cell, which yields actuation of tens of nanometres with sub-millisecond temporal resolution^18^, and also dynamic magnetic field remotely controlled apoptosis using SPIONs rotations^19^.

To develop effective cell therapy, the location, distribution and long-term persistence of transplanted cells must be evaluated and microscopically monitored. Magnetic labelling of nonphagocytic cells had been mainly conducted with the FDA-approved SPIONs Feridex (dextran coated) and Resovist (carboxydextran coated)^19–22^, which makes the cells detectable by magnetic resonance imaging (MRI)^23–25^. These, as well as other SPIONs, were coated with negatively charged molecules to make them hydrophilic and avoid particle aggregation. However, this coating leads to electrostatic repulsion between the nanoparticles and targeted cells, since both have negatively charged surfaces, which causes difficulties in nanoparticle delivery^20–23^.

The main limitations to real time *in-vivo* applications of both the techniques thus may be attributed to the lack of, (a) an efficient and remotely controlled dynamic process for electroporation on a single targeted cell; (b) a mechanism to perform accurate transporting (replacement or repositioning) of modified cell, and (c) a family of probes to track and monitor the to-be-replaced or newly-modified cells in both cell electroporation and cell therapies. In this work, we have reported that magnetoelectric core-shell structured nanoparticle composites have been developed to overcome all three complexities and perform functions as magnetoelectric nanorobots (MENRs) under magnetic field excitation for single cell manipulations. The MENRs are individual magnetoelectric nanoparticles with single crystalline ferromagnetic core of CoFe_2_O_4_ (CFO), exhibiting magnetostrictive property and thin film crystalline ferroelectric shells of BaTiO_3_ (BT), exhibiting piezoelectric property. A MENR works for targeted single cell electroporation (via localized periodic e-pulses generation and e-field sensing), or for replacement and repositioning of new or modified cells (via thrust generation), remotely controlled by magnetic field in ways described as follows.

Firstly, when exposed to an a.c. magnetic field (H1) of 60 Hz in frequency and 50 Oe in magnitude, an MENR acts as a localized periodic electric pulse generator. Under this a.c. magnetic field excitation, CFO core of MENR experiences magnetostriction and generates elastic waves along with a rotational attractive magnetic moment towards the field. These elastic waves are absorbed by the piezoelectric BT shell that in-turn generates highly localized periodic electric pulses with maximum intensity of 10.25 μV/nm*Oe and at 8.33 millisecond period^26^. When human epithelial cells (HEP2) were introduced to the vicinity of these negative periodic pulses at sub-micron distances, the phospholipid on cell membrane dislocates temporarily due to electrostatic repulsion of phosphate head (PO_4_^3−^). The lipid bilayer do not reseal itself in between each of applied e-pulse duration (ON time), due to high comparative relaxation time of the phospholipid bilayer^27,28^. This in turn creates temporary defects in the form of nanopore on cell membrane. The nanopore on cell membrane increases in size and allows MENR to penetrate through it into the cell, upon its forward rotational magnetic moment caused by the H1 a.c. magnetic field. This mechanism of electrically excited cell permeation is named magneto-elasto-electroporation (MEEP)^26^.

**Figure 1:**
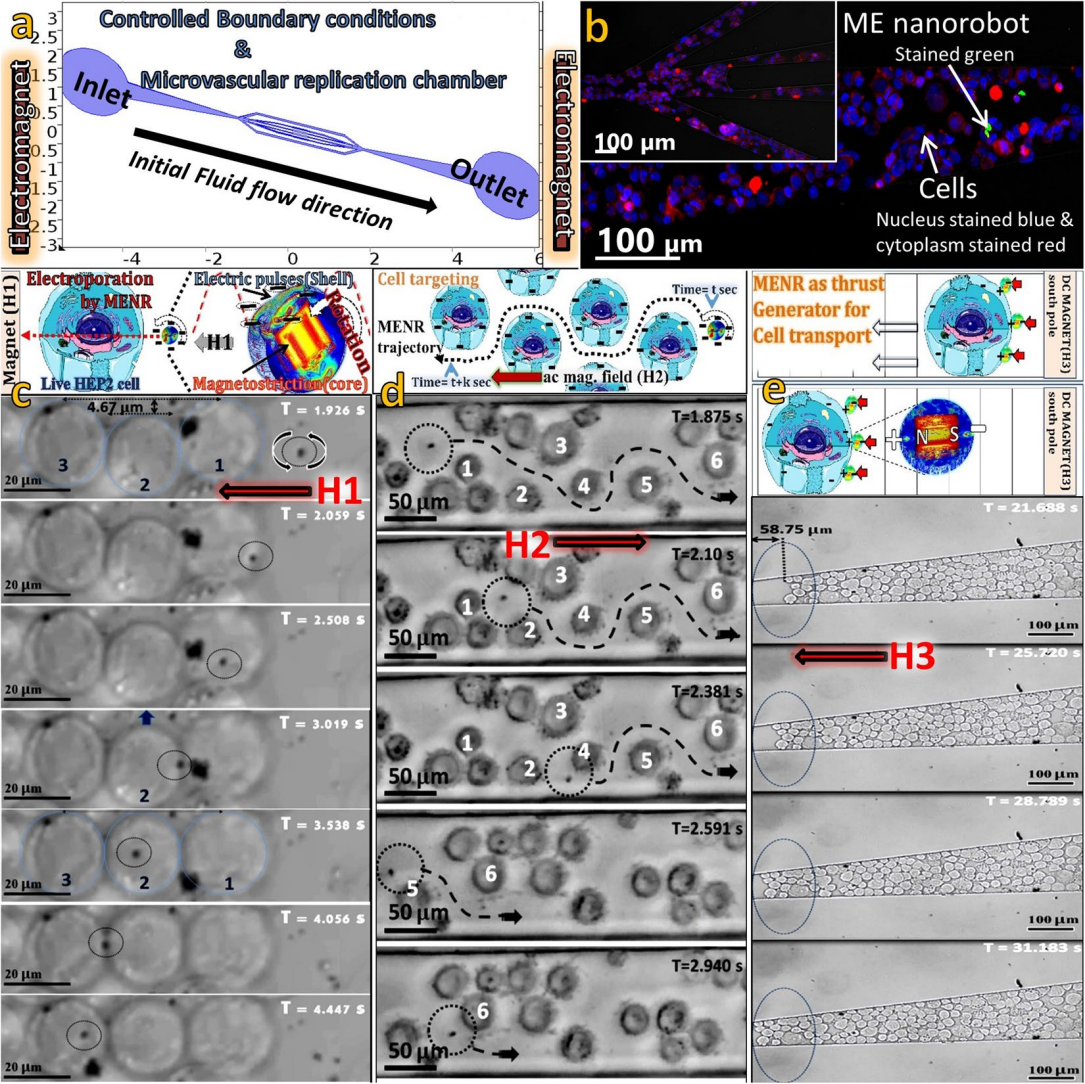
ME nanorobot - cellular interaction under remotely applied magnetic field excitation. (**a**) COMSOL model of the MSMC to perform MENR - cellular interactions in controlled boundary conditions. (**b**) Hep2 cells with nucleus (stained blue) and plasma membrane (stained red) present in the thickest area near outlet of MSMC along with MENR (stained green) and in the complex bifurcation area (see inset). Schematics and experimental time-points of (**c**) MEEP phenomena of cell permeation using MENR under influence of a.c. magnetic field (H1 = 50 Oe & 60 Hz); (**d)** Cell targeting by ME nanorobot to an area avoiding any of the cell on pathway via electrostatic repulsion under influence of a.c. magnetic field (H2 = 40 Oe & 30 Hz); and (**e)**, Cell transported by group of MENRs under influence of d.c. magnetic field (H3 = -50 Oe). Cells are pushed by MENRs inside microfluidic chamber to fill 58.75 µm of void space.

We have designed a custom build systematic setup (Fig. 1a) to accurately examine the MENR-cellular interaction at this particular magnetic field and performed experiments in microvascular structured microfluidic chamber (MSMC) with complex junctions and variable pressure gradient^29^. Electromagnets were attached to two ends of the MSMC. MENRs were added to the cell suspension and were inserted into the MSMC through the inlet towards the fluidic flow direction (Fig. 1a). Directional a.c. magnetic field and biased d.c. magnetic field were remotely applied subsequently from either ends of the MSMC to perform the experiments. Time-lapse videos were captured using fluorescent microscope in bright field mode at 10X, 20X and 40X magnifications for subsequent image processing. The inserted HEP2 nuclei were further stained with DAPI (4’,6-diamidino-2-phenylindole) - blue dye and plasma membrane were stained with CellMask – deep red dye; and MENRs were coated with silica and stained with FITC (Fluorescein isothiocyanate) - green dye, to confirm the presence of the MENRs and cells inside MSMC via fluorescent microscope images (Fig. 1b).

These experiments have revealed that while cell permeation at H1 field excitation, MENR also excites inter-cellular processes such as signalling, communications and self-patterning. A single MENR permeates inside HEP2 cell can cause a group of cells to align themselves in an equipotential mono-array to form a pathway of the MENR with the lowest impedance. A time-lapse microscopy video of this phenomenon (at 40X magnification) is presented in supplementary movie (Mov. 1). A few different time-points of supplementary movie (Mov. 1) are presented in Fig. 1c, where cell permeation, inter-cellular signalling and cell electromechanical motion induced by MENR, under influence of H1 a.c. magnetic field can be observed. Cells marked 1, 2, 3 (Fig. 1c) are nearby cells among which Cell 2 is 4.67 µm off-aligned initially from its neighbours. MENR highlighted in dotted lines could be seen rotating and moving forward under the influence of H1 a.c. magnetic field against the initial fluid flow direction, which was used to fill the MSMC (Fig. 1a). Upon the penetration of MENR into Cell 1, and as it moves inside Cell #1 and closer to Cell #2, Cell #2 aligns itself by moving upward to provide lowest impedance pathway. Cell #2 holds its position immediately allowing for the penetration of MENR, and MENR enters Cell #3. MENR is at the junction of Cells #2 and #3 but there is no further motion observed in either of the cells since they all are at equilibrium impedance position. It can also be seen in the same region of interest that a clump of nanoparticles does not enter the cells and do not produce the same effect as crystalline shell single nanoparticle.

Secondly, when exposed to an a.c. magnetic field (H2) at comparative lower frequency and amplitude (40 Oe & 30 Hz), applied from outlet end of MSMC, these MENR can be targeted to any area, a particular cell or distances irrespective of the cell medium density, and avoiding any of the cells on its path. The cause of such MENR-cellular interaction is that, MENR still generates periodic negative pulses and undergoes rotational attractive magnetic moment under H2 magnetic field conditions. But the intensity of electric pulses and frequency of pulse repetition is low as compared to H3 field excitation. When close to cell vicinity under H2 field excitation, the periodic electric pulses generated by the MENR have ON-interval greater than phospholipid bilayer relaxation time^27,28^. Hence, the applied electric pulse is incapable of dislocating the phospholipids on cell membrane. Instead MENRs experiences electrostatic repulsion from the cumulative negative charge of the phosphate heads on cell membrane. This repulsion leads to avoidance of any cellular interaction on the pathway and also the MENR gain speed in high cell density area due to higher cumulative electrostatic repulsion but never interacts with any of the untargeted cell. Hence, MENR can be targeted to any cellular density area as well as to a single cell by switching off the electromagnets, when MENR reaches the target. The mechanism is experimentally validated and visual confirmations via time-lapse video microscopy at 20X magnification are reported in supplementary movies (Mov. 2). The schematics of cell targeting and different time points of supplementary movies (Mov. 2) are presented in Fig. 1d. MENR is moving forward without interaction with any of the cell #1–6 on its pathway and accelerates when comes closer to the cellular negative field flux. This proves that MENR acts as a nanoprobe which can sense the negative electric potential of a single cell or a group and is repelled to lowest electric flux areas under H2 field excitation. We have also observed that large clumps of nanoparticles do not exhibit this phenomenon.

Thirdly, when d.c. magnetic field (H3) of intensity −50 Oe, is applied through outlet end of MSMC, MENR’s ferromagnetic core instantly experiences unidirectional strain. This induces electrical dipole on piezoelectric shell in preferred directions such that the negative end of the electrical dipole always directs towards the south pole of the magnetic core, verified in the later part. When the MENRs are in close vicinity of the huge negative suspended cells and subjected to H3 field, the instantly created electrical dipole’s positive head (core’s magnetic north pole) attracts and attached with ionic bonds to the negative phosphate heads on cell membrane. The magnetic south pole of the MENRs face towards the −50 Oe d.c. magnet (south pole) and experiences continuous repulsion. Since the MENRs are attached with the suspended cells and continuously exposed to the H3 field, they act as a thrust generator and steers the cells controllably to indefinite distances, until they experience blockage on the path or magnetic field intensity decreases or switched off. Experiments on MENR-cellular interaction at H3 field excitation were performed in MSMC and recorded real-time video at 10X magnification of this process is presented in supplementary movies (Mov. 3). The schematics of cell actuation and different time points of supplementary movies (Mov. 3) are presented in Fig. 1e. Experiments also confirm that MENR can uniquely actuate suspended cells without disturbing any of the adhered cell area as shown in supplementary movies (Mov. 4). MENR can actuate a group of live cells to a targeted area in controlled boundary condition of MSMC under (H3) magnetic field excitation and proves the capability to conduct replacement, positioning and patterning of modified cells in dehydrated or disconnected neural circuitry.

**Figure 2:**
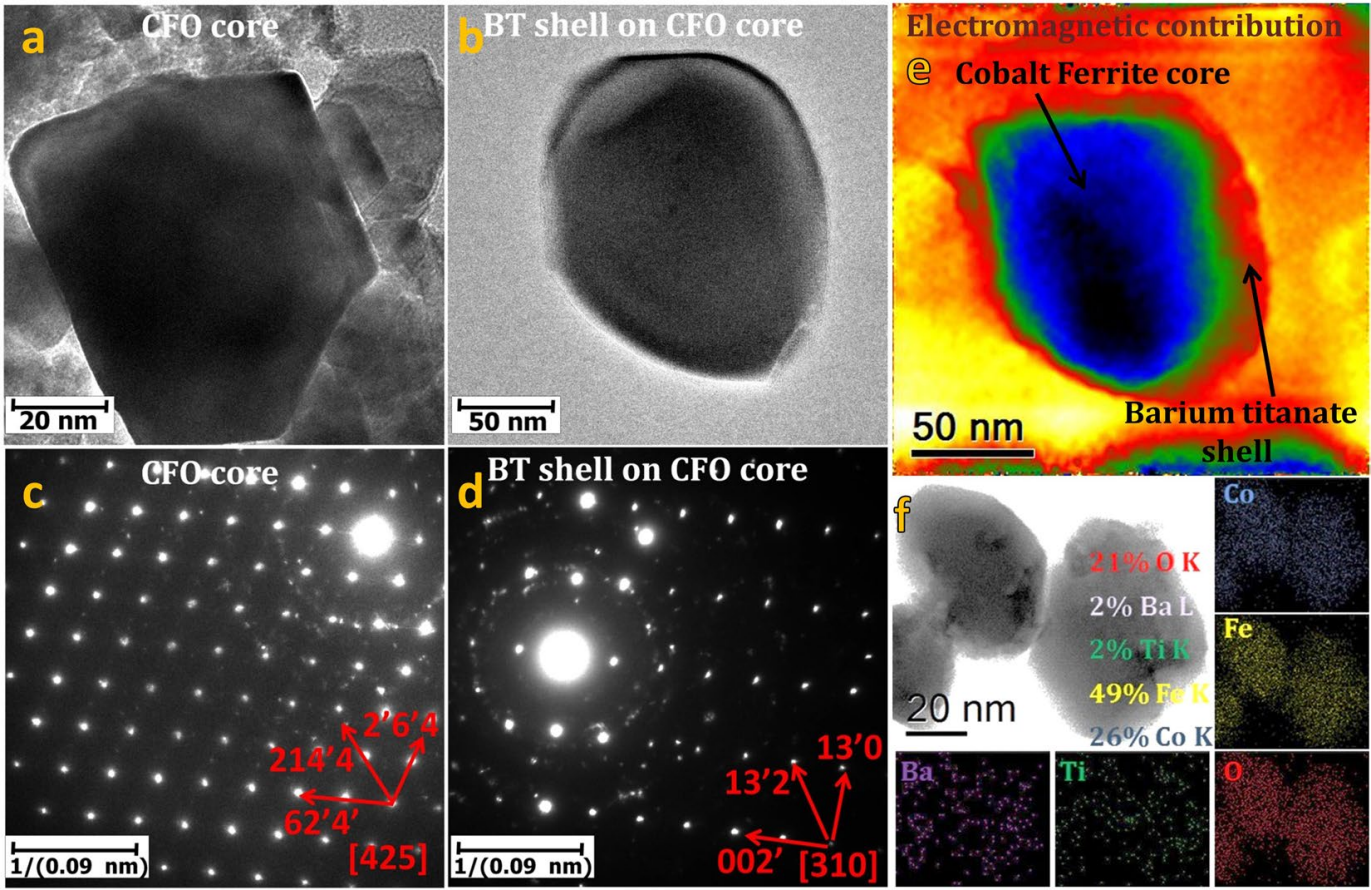
MENR microstructure characteristics and chemical composition. (**a**) TEM image of CFO core. (**b**) TEM image of MENR - BT shell coated CFO nanoparticle. (**c**) SAED pattern of crystalline CFO with zone axis <011>. (**d**) SAED pattern showing <$1\bar{1}\bar{2}$> zone axis for BT layer. EH image showing (**e**) MENR’s electromagnetic contribution reconstructed from phase extraction. (**f**) A BF-SEM image with the corresponding EDX element distribution mapping of three core-shell nanostructures along with atomic wt. (%) composition.

MENRs were fabricated using hydrothermal process discussed in method section. The core used is CoFe_2_O_4_ nanoparticles, obtained from (a) commercial source (Alfa Aesar Inc.). Microstructure images of the MENR were acquired using a transmission electron microscope (JEOL 2010F) working at 200 KV. The CoFe_2_O_4_ nanoparticles were uniform in shape and size with predominant cubic morphology as shown in Fig. 2a. The out of focus image (Fig. 2b) to distinguish CFO core after (a) uniform coating of BT was achieved. The deposition process is described in detail in the Materials and Methods section. To substantiate the crystalline nature of the core and shell, selected area electron diffraction (SAED) patterns were recorded and indexed with JEMS-EMS java version (see supplementary information, Fig. S1). The diffraction patterns shown in Fig. 2c and d, substantiates the crystalline nature of the CFO and BT correspondingly. The electromagnetic contribution of the nanostructure (Fig. 2e), when excited by 1.5 Tesla magnetic field of objective lens (Holo-mode), was extracted using off-axis electron holography (EH) and the technique is discussed in details in the later part and supplementary information. Clearly there are visible distinctions between the electromagnetic contributions of the core-shell bi-phasic nanostructure as shown in Fig. 2e. To establish the allocation of elements through the core-shell nanostructure, energy dispersive X-ray analysis (EDX) mapping was performed on an area of the grid having three core-shell nanostructures at the region of interest as shown in Fig. 2f, which displays a bright-field (BF) image obtained by scanning electron microscopy (SEM). The elementary distribution (Fig. 2f) is confirmed by EDX mapping which shows the element position with atomic weight percentage as barium (violet) - Ba L (2%), titanium (green) - Ti K (2%) (from BaTiO_3_) are particularly confined at the shell, whereas cobalt (blue) - Co K (26%), iron (yellow) - Fe K (49%)- (from CoFe_2_O_4_), rich zones are observed at the core. Oxygen (red) - O K (21%) is distributed throughout the nanostructure. Semi-quantitative energy dispersive X-Ray analysis (EDXA) data presented in supplementary information (Fig. S2), also corroborates the elemental distribution in the core and shell of the nanostructure. For electrical property analysis of MENR, piezo-response force microscopy (PFM) was performed and presented in supplementary information (Fig. S3). PFM samples were prepared as discussed in Material and Method section. The Piezo-response force microscopy measurements were performed with a tip biased voltage of ±1 V, ±2 V and ±10 V on the nanoparticles. PFM results clearly shows polarization state switching capability of the individual MENR due to the converse-piezoelectric effect, as tip voltage changes from positive to negative biased voltage intensity. This result provides clear evidence that the BaTiO_3_ coating is ferroelectric in nature and underlies its tetragonal perovskites phase at room temperature. Moreover, polarization states may be fully controlled by electric potential and no poling is required due to crystallinity of shell. Any pressure generated by strain of the CFO core, will be transferred to the BT shell and there will be surface potential change on the BT coating. This transfer of mechanical wave from core to shell in the core-shell structure is also confirmed through optoacoustic measurements as discussed in supplementary information (Fig. S4).

**Figure 3:**
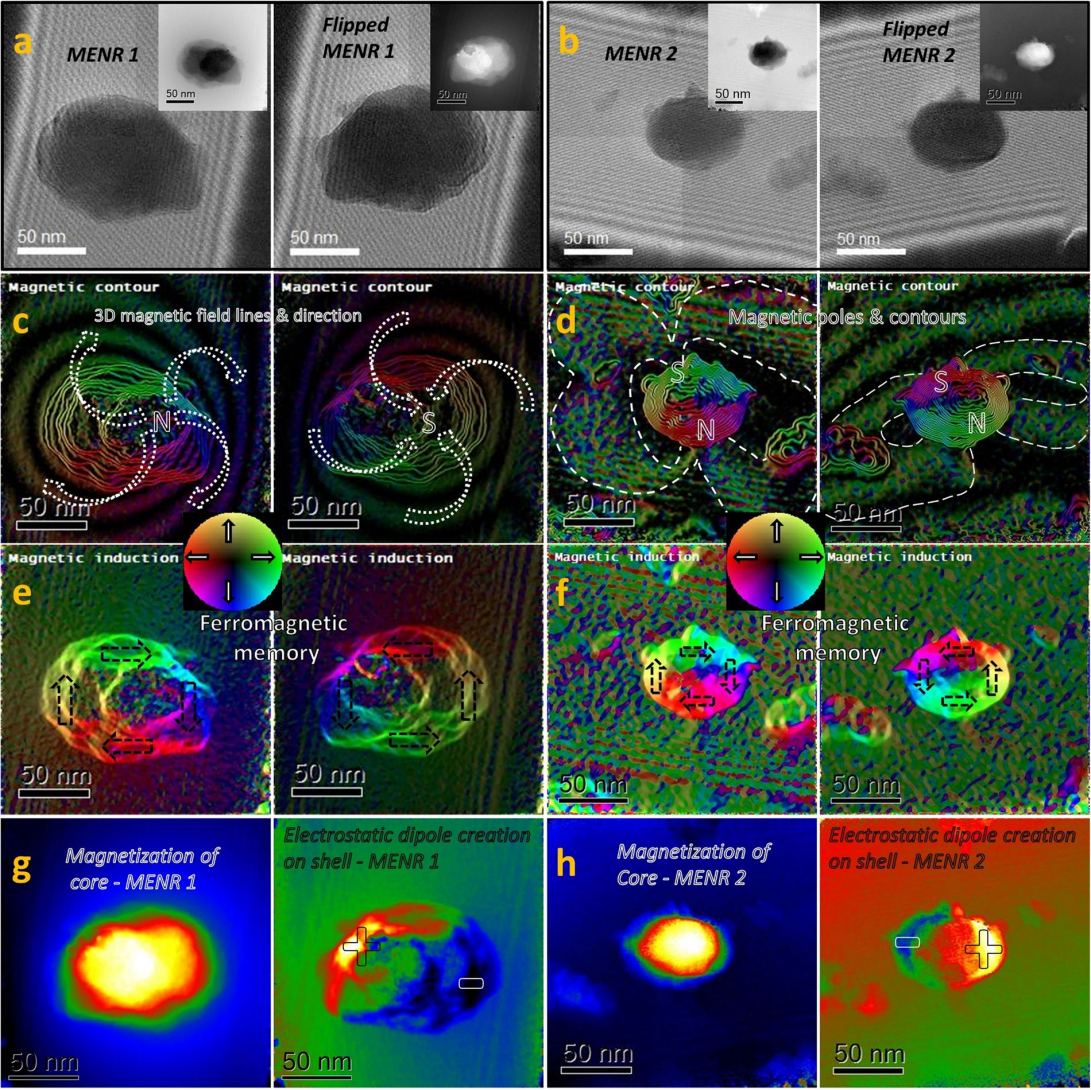
Electromagnetic characterization of single MENR using EH. (**a**) EH of the un-flipped (left panel) and 180° flipped MENR1 (right panel), with extracted phase image (see insets). (**b**) EH of the un-flipped (left panel) and 180° flipped MENR2 (right panel), with extracted phase image (see insets). (**c**,**d**) Magnetic contour (in colour) of the amplified phase (the cosine of the magnetic phase has been amplified four times in case of MENR 1 and eight times in case of MENR2 for both un-flipped (left panel) and flipped (right panel)) to see the magnetic pole formation and magnetic field line envelop. MENR1 is oriented ⊥ to MENR 2 in 3-D space. (**e**,**f**) Ferromagnetic memory in form of magnetization direction is retained from un-flipped to flipped MENR 1 and 2. (**g**) and (**h**), Magnetization concentrated on the core (left panel) and electrostatic surface potential (right panel) concentrated at the shell of MENR 1 & 2. Electrostatic dipole formation on shell (right panels) created in preferential direction of magnetic poles.

Ferromagnetic property of the core contributing to the rotational motion under fluctuating field excitation, electrical dipole creation on the shell by d.c. magnetic field, and magnetization of the MENR have been analyzed using off-axis electron holography on a TEM JEOL ARM200F, a method reported by researchers^30,31^. From previous results, we have measured the residual magnetic field on the microscope (around 50 Oe) and the induced EM field as a function of the condenser lens excitation (up to 2 T in HR-TEM)^31^. The holograms have been acquired using a Fresnel fringe contrast of 23%, which was adjusted with a bias voltage of 40 V applied to the biprism attached to the microscope. For the registered holograms, an exposure time from 2–4 s was used to enhance the phase resolution. The holograms were recorded with Gatan’s Digital Micrograph (DM) software, and reconstructed by a beta version of HoloWorks 5.0.7. Every retrieved phase was numerical reconstructed using a reference hologram and the object hologram to remove the influence of the unperturbed reference wave. The phase separation between the electrostatic potential and the magnetization of the MENR is extracted by flipping (up and down) the sample manually and the procedure is described in details (see supplementary information, Fig. S6). Magnetic and electric properties of 2 of MENRs situated in different orientation were analyzed in our experiment, where one is oriented laterally at an acute angle in perpendicular direction of the interference fringes (Fig. 3a (left panel)) and the other is parallel to the Fresnel fringes (Fig. 3b (left panel)). These 2 MENRs were manually flipped 180° as shown in (Fig. 3a and b, (right panels)) and were analyzed using EH. The magnetization of MENR in response to the 1.5T magnetic field of the objective lens (on Holo-Mode) during EH, enhances the magnetic pole intensity on the two shown MENRs. These 2 MENRs are oriented perpendicular to each other, in such a way that, in one case we can only see one of the magnetic pole, contour and 3-D magnetic field lines with direction (Fig. 3c (left panel)), whereas in other case we can see both the poles and the magnetic field lines envelope formed between the magnetic poles (Fig. 3d (left panel)). On flipping both the samples the magnetic contours orientation is shifted and can be clearly seen in (Fig. 3c and d, (right panels)). These magnetic contours were obtained by amplification of the phase to 4 times for MENR1 and 8 times in case MENR 2. The pole formation on MENR was also confirmed by magnetic force microscopy (MFM) measurements (see supplementary information, Fig. S5). While analyzing the magnetic induction in un-flipped sample (Fig. 3e and f, (left panels)) and flipped sample in (Fig. 3e and f, (right panels)), though the excitation magnetic field direction is same and there is 180° flipping in the sample, but the direction of magnetization of MENR in (Fig. 3e and f, (left panels)) i.e. counter-clockwise remains in the same direction, even after flipping i.e. magnetization direction is now clockwise in (Fig. 3e and f, (right panels)). This shows that the MENR behaves as permanent magnets, a phenomenon attributed to the ferromagnetic memory of CFO core. Figure 3g and h, (left panel) shows the magnetization contribution and Fig. 3g and h, (right panel) shows the electrostatic surface potential flux contribution, which have been extracted from the subtraction and addition respectively, of the un-flipped and flipped MENRs phases. In (Fig. 3g and h), following observations can be clearly seen in the magnetization contribution and electrostatic surface potential contribution of MENR 1 and MENR2, when excited by 1.5 T magnetic field of the objective lens on Holo-Mode: (a) The magnetization flux is concentrated on core since the size of the MENRs are observed small in magnetization contribution image (Fig. 3g and h, (left panels)), as compared to the electrostatic surface potential contribution image (Fig. 3g and h, (right panels)), where the size of the same MENRs seems comparatively larger, as electrical flux is concentrated on shell only. (b) Electrostatic dipole formation on the shell of the MENR 1 and 2 when excited by 1.5 Tesla magnetic field of the objective lens during EH. The electrical dipole formation have preferred orientation on MENR’s shell i.e. negative electric pole on shell originates toward magnetic south pole of the core and positive electric pole of the shell towards magnetic north pole of the core, which can be seen by comparing Fig. 3c (right panel) with g (right panel) and Fig. 3d (right panel) with h (right panel).

By subtracting the phase map so obtained from the un-flipped and flipped phase maps. The total phase shift (ΔΦ) across the MENR1 in the unwrapped phase image is half of {30 − (−22) = 52 radians} and across the MENR 2 is half of {15 − (−9) = 24 radians}, see supplementary information (Fig. S6). A quantitative evaluation of the magnetic flux can be obtained from its constructed phase maps since between two adjacent contour lines, a magnetic flux of $\bar{h}/e$ = 4.1 × 10^−15^ Wb is included, thus a phase difference of 2π corresponds to a magnetic flux quantum of $\bar{h}/e$ ^31^. The total phase shift (ΔΦ) across the nanoparticle in the unwrapped phase image, is proportional to the total flux by equation (1), thus the magnetization can be evaluated,

1 $${\bf{M}}=\frac{{\boldsymbol{\Delta }}{\boldsymbol{\Phi }}\ast {{\boldsymbol{\phi }}}_{0}}{2{\boldsymbol{\pi }}\ast {\mu }_{0}\ast {\bf{S}}}$$

Where, S is the cross-section area of the nanoparticle, µ_0_ is the magnetic permeability of the vacuum and φ_0_ is the magnetic flux quantum ($\bar{h}/e$). Assuming the shape as a prolate spheroid, the cross-section area for MENR 1 and 2, calculated by Knud Thomsen’s equation are approx. 3.28 × 10^−14^ sq. meter and 1.06 × 10^−14^ sq. meter (see supplementary information (Fig. S7)). The saturation magnetization (M_sat_) induced in MENR 1 and 2 due to 1.5 Tesla magnetic field of objective lens (Holo-mode), so obtained from eq. 1 are 0.412 × 10^6^ A/m and 0.586 × 10^6^ A/m respectively. The measured M_sat_ of MENR through EH was higher than the experimental bulk M_sat_ which is approx. 0.236 × 10^6^ A/m as measured from the bulk ferromagnetic hysteresis data (see supplementary information, (Fig. S8)). The possible reason of this minute mismatch is: firstly, there is still uncorrected residual electric contribution because the holograms where not acquired in lorentz conditions (objective lens off), due to complexities discussed in supplementary information (Fig. S9) and, secondly the density assumed in bulk magnetization calculation is the average density.

Lastly, MENR were observed bio-compatible as tested for cytotoxicity on HEP2 and NG-108 rat neuronal cells up to concentration of 50 µg/ml and 20 µg/ml, respectively in aqueous medium (see method section and supplementary information (Fig. S10)). The fabricated MENRs have a uniform deposition of perovskites (barium titanate) on spinel (cobalt ferrite) with expected high lattice mismatch, which is unique and never been reported. Since magnetic nanoparticles acts as a contrast agent for MRI, these MENRs can be easily tracked with high accuracy by using MRI imaging while performing *in-vivo* experiments. Implementation of real-time application of the MENR functionality for *in-vivo* experiments, and the proposed integrated device (MSMC, directional electromagnets and MENR) for *in-vitro* experiments, can revolutionize the field of study of electrical property of various live cell types, targeted single cell electroporation, targeted drug delivery and cell-based therapies.

The finding in this research is the first time ever elaborated visible proof with experimental confirmation and complete understanding of magnetoelectricity at nanoscale in an inorganic core-shell nanocomposite and its nanorobotics property in bio-cellular environment. These core-shell nanocomposites comprised of single crystalline ferromagnetic cores of cobalt ferrite coated with crystalline ferroelectric thin film shell of barium titanate. The importance of this research comprises of the innovation of developing a remotely controlled dynamic process for manipulation of targeted live biological cells using the spin orbital interaction of the magnetoelectric nanocomposites under biased and fluctuating magnetic field excitation. These nanorobots, controlled remotely by a.c. or d.c. magnetic fields, performs dynamic cellular manipulation which includes cell targeting, permeation, patterning and transport. MENRs performs these functions via localized electric periodic pulse generation, local electric-field sensing, or thrust generation and acts as a unique tool for remotely controlled dynamically targeted cellular manipulation. Further, elaborated microstructure analysis and chemical characterization were performed which shows that the fabricated MENRs have a uniform deposition of perovskites (barium titanate) on spinel (cobalt ferrite) with expected high lattice mismatch, which is unique and never been reported. This approach of nanorobotics in cellular environment have promising applications, since these nanorobots are the only tool for targeted single cell electroporation and targeted cell transport. These functionalities will simplify the current complex methods of single nanopore studies on cell membrane via electroporation and modified cell transport techniques for vascular repair. This multidisciplinary work will have a strong impact on the therapeutics field.

## Methods

### Microvascular structured microfluidic chamber (MSMC) fabrication

#### Mold Fabrication

Molds for microfluidic device were fabricated on Silicon wafers using photolithographic techniques. The molds were fabricated for 7 µm height. The first step was to treat the silicon wafers with piranha solution (a solution containing 2 parts H_2_SO_4_ and 1 part H_2_O_2_). The piranha solution was let to sit for 5 minutes after the appropriate volumes of the reagents were added. The silicon wafers were then dipped in piranha solution for 8 minutes. The silicon wafers are carefully removed and dried with Nitrogen. The drying step continued with baking the silicon wafers at 200 °C for 5 minutes. Once the silicon wafers are completely dry, they are spun coat with the photoresist SU-8 5 (MicroChem, Newton, MA, USA). Enough photoresist was added to cover around 70% of the surface and the silicon wafers were initially spun at 500 rpm for 5 seconds with acceleration of 100 rpm/sec. This was followed by a second spin cycle of 2000 rpm for 30 seconds with acceleration of 300 rpm/sec. The final spin step was at 500 rpm for 5 seconds with acceleration of 100 rpm/sec. After spin-coating, the silicon wafers underwent a soft bake process, initially at 65 °C for 2 minutes, followed by 95 °C for 5 minutes. This was followed by exposure to UV light to cause the cross-linking in the photoresist. The mask with the appropriate design (described above) was used to expose the required pattern. The silicon wafers were exposed to a hard type exposure for 25 seconds at a power of 7.5 and A.I. gap of 40. There was a post exposure bake initially at 65 °C for 2 minutes, followed by 95 °C for 3 minutes. The silicon wafers were then developed by first rinsing them in developer solution for around 1 minute. They were then washed using isopropyl alcohol and blow dried with Nitrogen. In case of incomplete development, or if there were white spots on the silicon wafers, the development steps were repeated. The final step was to hard bake the silicon wafers, initially at 65 °C for 1 minute, followed by 95 °C for 1 minute, and ended with baking at 200 °C for 2 minutes.

#### Polymeric microfluidic device fabrication and final device assembly & operation

The silicon mold was used to make polymeric micro-channels using Polydimethysiloxane (PDMS; Sylguard Elastomer Kit, Dow Corning, Midland, MI, USA). Crosslinker (included in kit) was added to PDMS in a ratio of 1:10. The mixture was thoroughly mixed till uniform viscosity was obtained throughout the volume. It is then degassed using a vacuum chamber till most of the bubbles are eliminated. The PDMS-crosslinker mixture is then poured on the silicon mold, and degassed again till most of the bubbles are removed (a few small bubbles are acceptable). It is then baked at ~50 °C overnight. Once the PDMS solidifies, the particular region containing a single device is cut with a surgical scalpel. The PDMS blocks with the embedded micro-channels were attached to clean glass slides (cleaned as per the earlier described protocol) using oxygen plasma treatment (Plasma cleaner PDC-32 G, Harrick Plasma, Ithaca, NY, USA). The device is then left to bake at ~50 °C for 1-2 hours in order to strengthen the bond. Silicon connector kits (PTFE tubing and head fittings, Dolomite microfluidics, Charlestown, MA, USA) were used so as to get better adhesion with PDMS. The channels were passivated by perfusing 5% HSA and washed with PBS. The procedure is described in literature^32^.

### Synthesis of core-shell magnetoelectric nanorobots (MENRs)

The Core-Shell Magnetoelectric nanoparticle composites were synthesized using hydrothermal methods. The CoFe_2_O_4_ nanoparticles used were obtained from commercial source (Alfa Aesar Inc.) Barium Carbonate (BaCO_3_) and Titanium Iso-propoxide (Ti(OCH(CH_3_)_2_)_4_) were mixed with citric acid in separate containers to obtain the Ba and Ti citrate solutions. For uniform coating of BaTiO_3_ on CoFe_2_O_4_ nanoparticles, these citrates were then mixed with CoFe_2_O_4_ nanoparticles in Ethylene Glycol and heated at 100 °C to paralyze the solution. To stabilize the barium titanate shell and achieve intended crystalline characteristics, the mixture is dried and heated further at 800 °C for 8 hour in a low supply of oxygen to prevent oxidation of the ferromagnetic nanoparticles. Finally, the dried powder was repeatedly washed using ethanol and de-ionized water and sonicated in ultrasound cleaner to obtain the final crystallized composites of BaTiO_3_ coated CoFe_2_O_4_ nanoparticles.

### FITC conjugation on Si Coated nanorobot

FITC was first conjugated to APTES. Typically, FITC (2 mg) was dissolved in 0.1 M APTES in ethanol. The solution was stirred in dark for 24 hour. FITC-APTES (5 ml) solution was then added to silica coated particles (10 mg) and was stirred vigorously for 1 hour. The solution was then incubated for 24 hour at 40 °C. The resulting solution was washed repeatedly by ethanol to remove un-conjugated FITC. The FITC loading on silica coated MENRs was confirmed using spectrophotometer measurement and results are shown below.

### Nucleus staining using DAPI dye

HEP2 Cell concentration for staining: 1 × 10^6^/ml. DAPI Conc.: (1:100 in PBS). DAPI dye was obtained from Thermo Fisher Scientific and staining was performed using the manufacturer’s protocols given in catalog# D1306. Cells were incubated at room temperature for 15 minutes with gentle rocking. This is followed by 3 washes with PBS and 1 wash with water. Each washing step was for 15 minutes at room temperature with gentle rocking.

### Plasma membrane staining using Cellmask dye

CellMask deep red plasma membrane stain was obtained from Thermo Fisher Scientific and staining was performed using the manufacturer’s protocols given in catalog# C10046.

### Sample preparation for AFM and PFM measurements

AFM and PFM measurement on nanoparticles is very complex since we need a substrate with atomically smooth surface (where surface roughness is very low/lower that the particle size-in nanometers). Moreover, particles must stick to the surface of the substrate and should be immobilized so that Voltage can be applied using PFM tip and tip can scan the nanoparticles at the same spot as it was on each scan. As shown in literature, to achieve this we use a Mica substrate and cleaved its surface multiple times (4–5 times) by using adhesive tape. With this process, we prepare atomically smooth surface. Then this cleaved mica substrate was carefully immersed in a mixture of (1:5) Poly-L-Lycin and DI water for 25 mins. This process will make the Mica substrate surface positively charges. Since nanoparticles have negative zeta/surface potential (discussed in earlier part), the nanoparticles stick to the surface of Mica and remain immobilized. Thus, both AFM and PFM scanning can be done efficiently.

## Electronic supplementary material

Supplementary movie (Mov.1)

Supplementary movies (Mov.2)

Supplementary movies (Mov.3)

Supplementary movies (Mov.4)

Supplementary information
