# Supplementary material for: Core-shell magnetoelectric nanorobot – A remotely controlled probe for targeted cell manipulation
Source: Sci Rep. 2018 Jan 29;8:1755. doi: 10.1038/s41598-018-20191-w (PMC5788862; doi:10.1038/s41598-018-20191-w)
Supplement: Supplementary file 5 — Supplementary information [file 41598_2018_20191_MOESM5_ESM.pdf]

Supplementary Materials

Core-shell magnetoelectric nanorobot – A remotely controlled probe for targeted cell manipulation

Soutik Betal[1]*, Amit Kumar Saha[2,3], Eduardo Ortega[4], Moumita Dutta[1], Anand Kumar Ramasubramanian[2,3], Amar Singh Bhalla[1] and Ruyan Guo[1]

[1] Department of Electrical and Computer Engineering, University of Texas at San Antonio, San Antonio, TX 78249, USA.

[2] Department of Biomedical Engineering, University of Texas at San Antonio, San Antonio, TX 78249, USA.

[3] Department of Biomedical, Chemical, and Materials Engineering, San José State University, San José, CA 95192, USA.

[4] Department of Physics and Astronomy, University of Texas at San Antonio, San Antonio, TX 78249, USA.

***** Corresponding author: email: soutik.betal@utsa.edu, soutik.betal2012@gmail.com

Selected area electron electron diffraction (SAED) pattern and indexing using JEMS software.

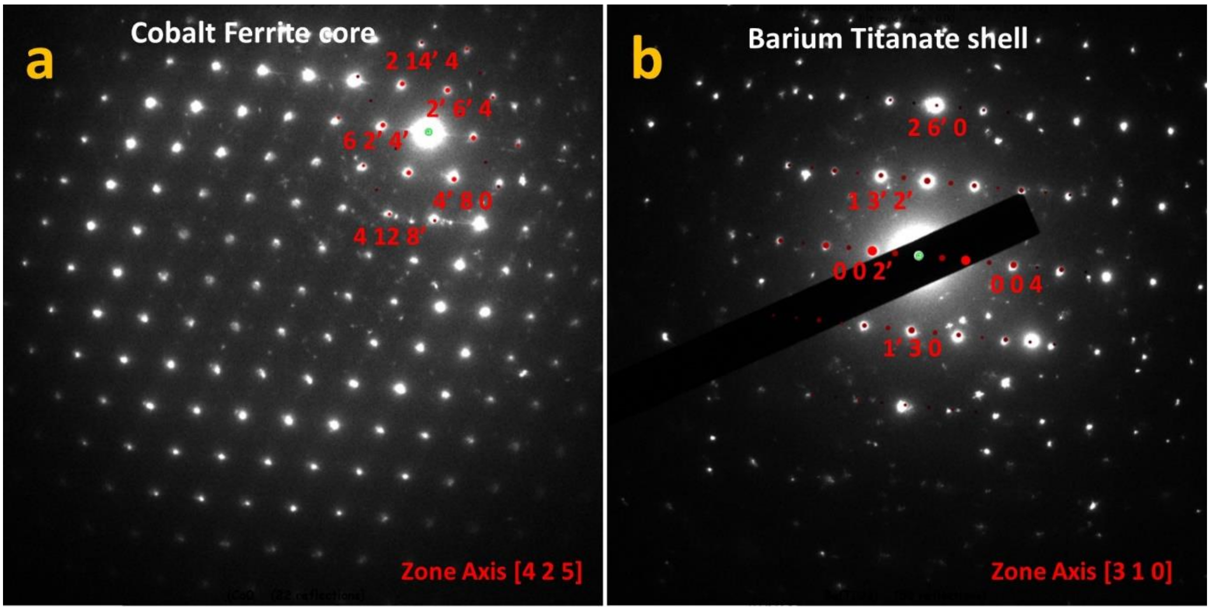

Figure S1 | **a,** SAED pattern of cobalt ferrite core with zone axis [4 2 5], and **b,** SAED pattern of barium titanate shell with zone axis [3 1 0]

Selected area Energy dispersive X-ray analysis (EDXA)

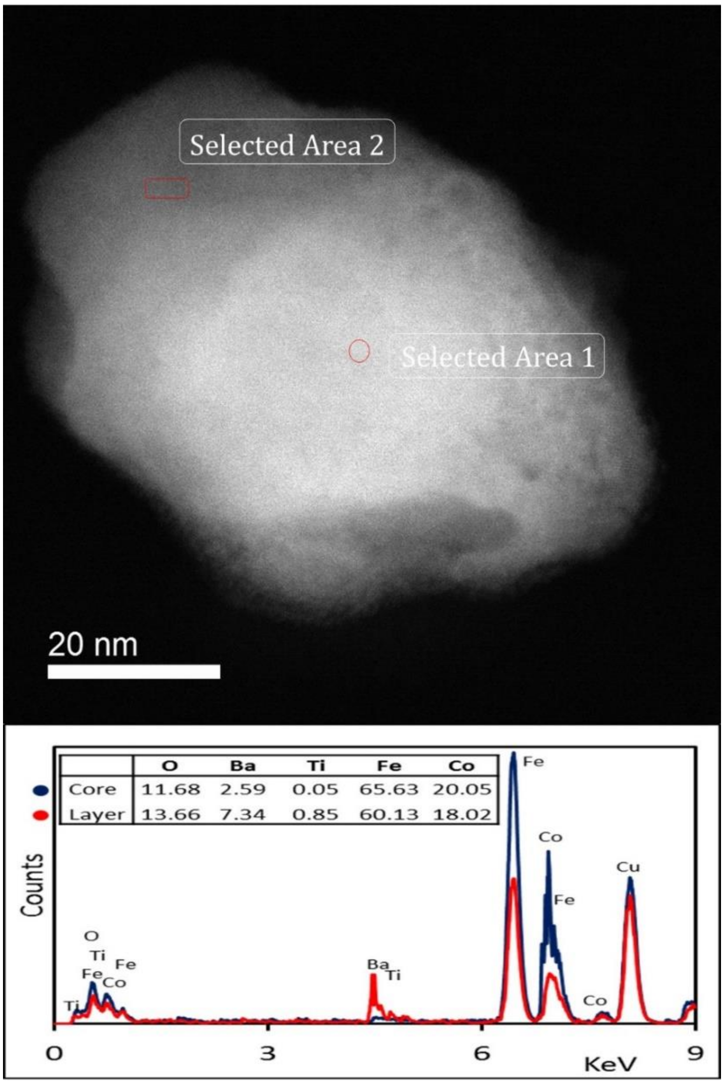

Figure S2 | Percentage composition of Co, Fe, Ba, Ti, O in the selected area of $BaTiO_3$ shell and coated $CoFe_2O_4$ core of the MENR.

Piezo-response force microscopy (PFM) measurements and Atomic Force Microscopy image

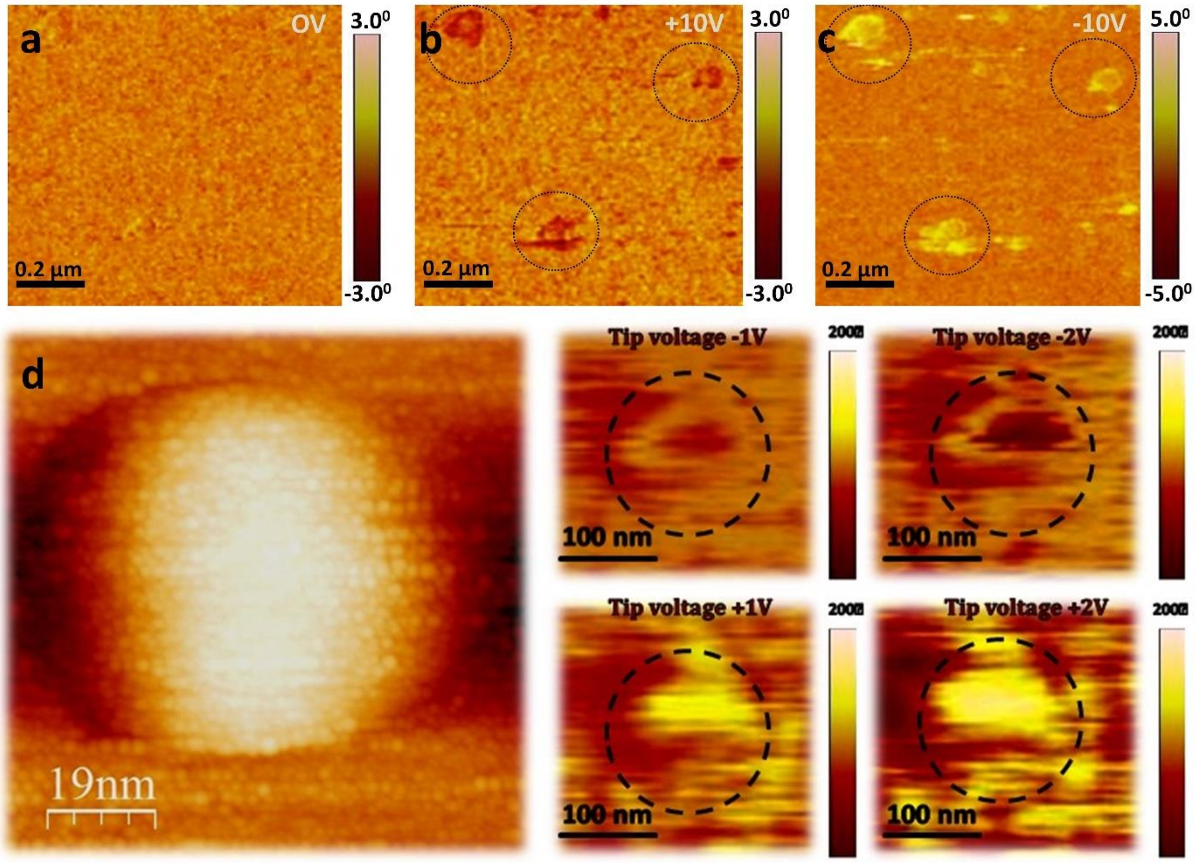

Figure S3 | PFM measurements performed at scanning mode with tip voltage [26] of **a,** 0V **b,** 10 V **c,** -10 V shows the phase switching behaviour with max phase change of -3° to 5°. **d,** Atomic force microscopy image of a single MENR and the high phase switching behaviour from -200° to 200° as measured by PFM with tip voltage of ±1V and also the enhanced phase and switching at ±2V.

Confirmation of transfer of stress from core to shell via Opto-acoustic measurements

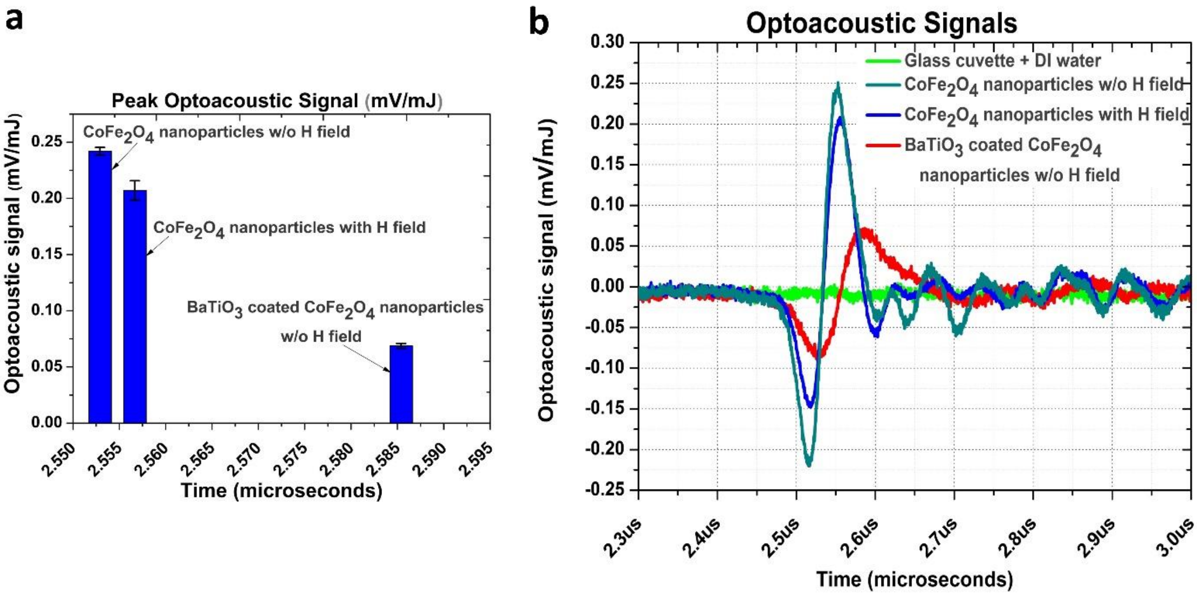

Figure S4 | Opto-acoustic Emission data and response curve [26]. **a,** Photoacoustic emission peak intensity of cobalt ferrite nanoparticles decrease when AC Magnetic field is applied and further when CSMEN were analysed the OA intensity peak further reduces. **b,** Opto-acoustic response curves of reference, CFO nanoparticles and CSMEN

Magnetic Force Microscopy (MFM) measurements images

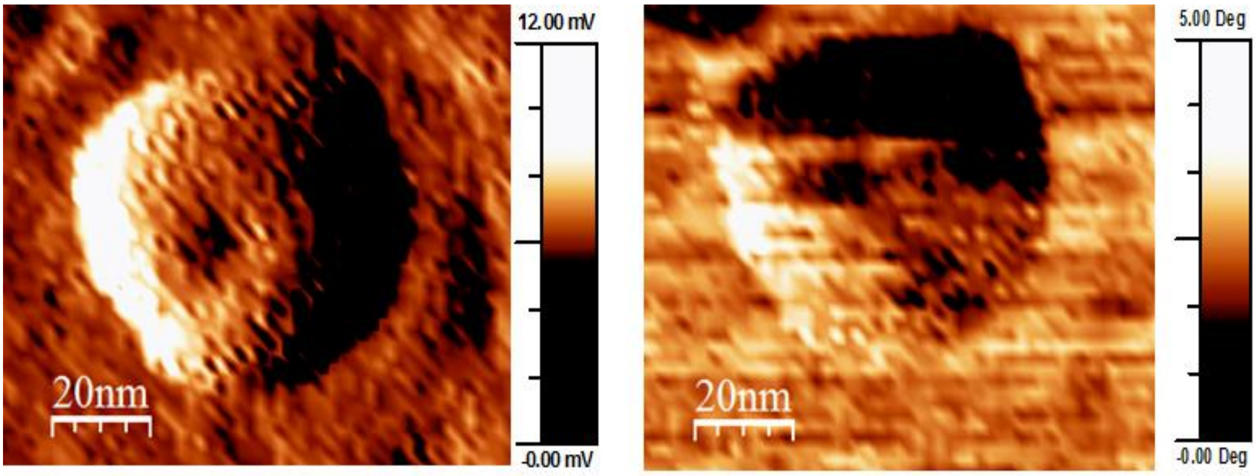

Figure S5 | Magnetic Force Microscopy (MFM) measurements images [33] over an area of 100nmX100nm: (left panel) Amplitude, (right panel) Phase. The Amplitude and Phase

Images represent the amount of shift brought about to the intial Amplitude and Phase to which the tip was tuned to, by the magnetic interaction between the Magnetic Domains of the particle with that of the magnetized Tip.While the darker regions signify attraction, the brighter regions is suggestive of repulsion. The amplitude image shows the pole formation on nanoparticles.

Elaborated description of electron holography measurements and analysis technique

Workflow to obtain unwrapped phase images from the electron holograms acquired. Figure presents the work flow to retrieve the phase information from the holograms using HoloWorks 5.0

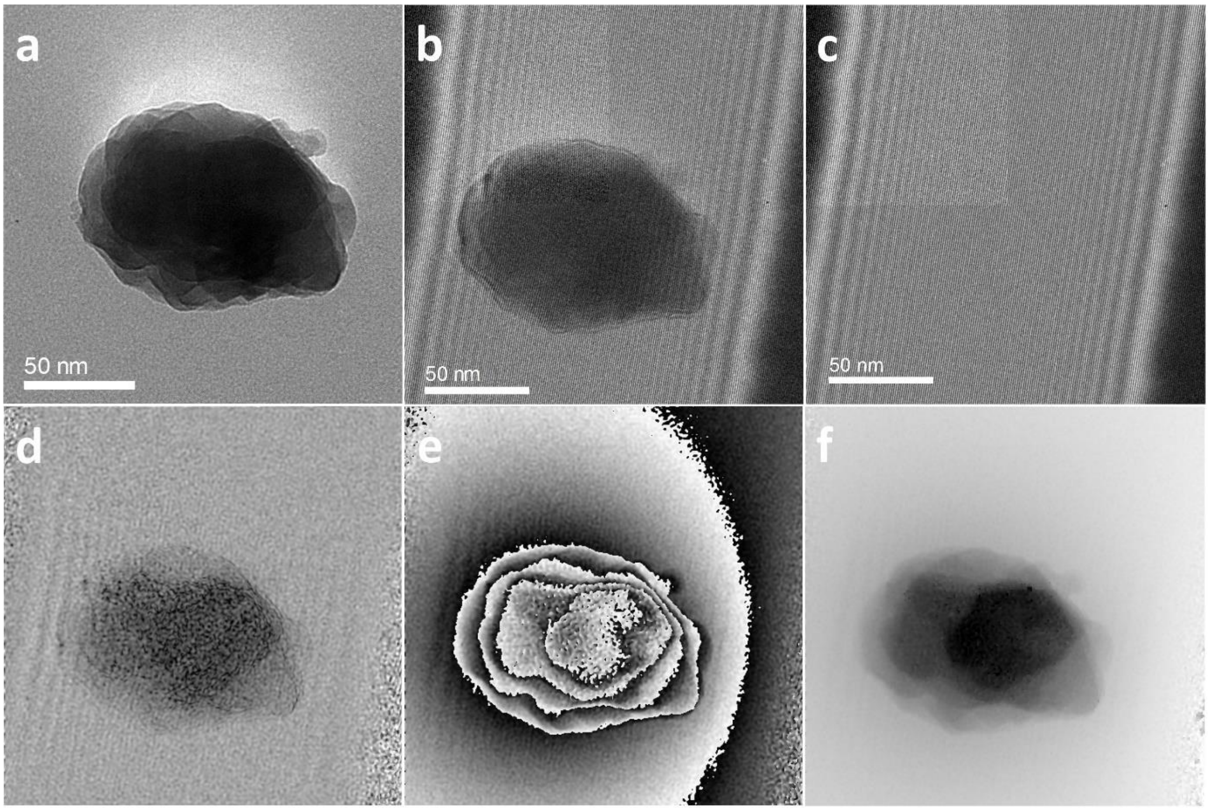

Figure S6 (i) | a, Region of interest on TEM. **b,** Electron hologram of the particle showed previously, the field of view is 165 nm. **c,** Reference hologram acquired with a fringe contrast of 23% and a biprism voltage of 40V. **d,** Complex image obtained from the inverse of the Fourier transform of the electron hologram where a circular mask is performed on one of the

sidebands. **e,** Modulo 2π phase image from the complex image. **f,** Phase unwrapping algorithms are used to remove the 2π phase discontinuities.

Off-axis electron holography details: The phase image obtained by the electron holograms possess information of not only the magnetic properties but also mean inner potential, for that reason is mandatory to separate these components to obtain reliable quantitative measurements. There are 4 ways to achieve it being turning the specimen over after acquiring a hologram and acquiring a second hologram from the same region the one utilized in our experiments.

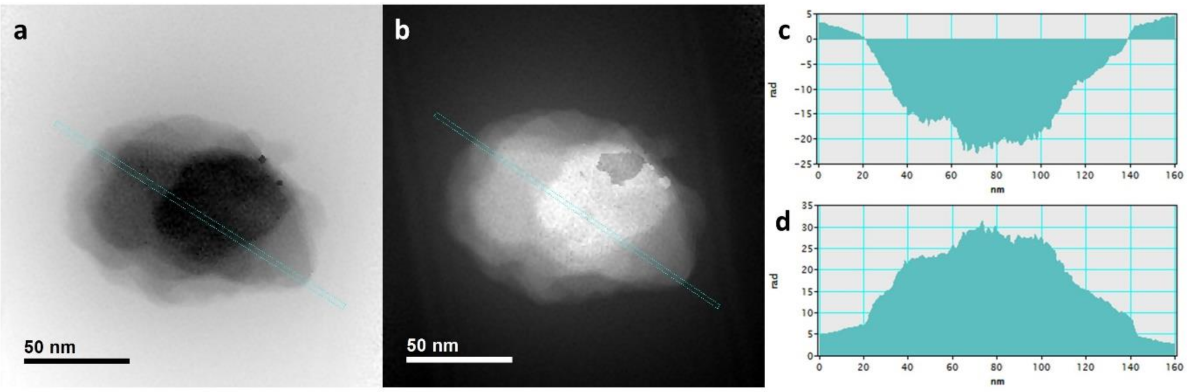

Figure S6 (ii): a, b, Phase reversal observed on a core shell nanoparticle when it has been flipped presents the phase reversal of a single core-shell particle. After flipping the microscope grid and taking a hologram from the same particle its phase image can be recovered. The phase inversion change can be observed as the contrast in the image is different. **c and d,** Moreover a line profile in the 2 phase images reveal that while the amorphous carbon (aC) layer maintains its positive value (around 4-5 radians) the particle passed from -23 to 30 rad, if we consider that the aC layer contribution is constant it will give a value of around -27 and 26 radians (subtracting on both cases the aC positive contribution).

Comparison of the behavior of a non-magnetic particle when applying the flipping procedure, an inversion is not produced. Let us compare the difference between a core shell nanoparticle (with a magnetic core) and a non-magnetic $BaTiO_3$ nanoparticle. Its holograms were recorded at the same time than the other nanoparticles. From this specimen we can't observe a change in the phase of the material.

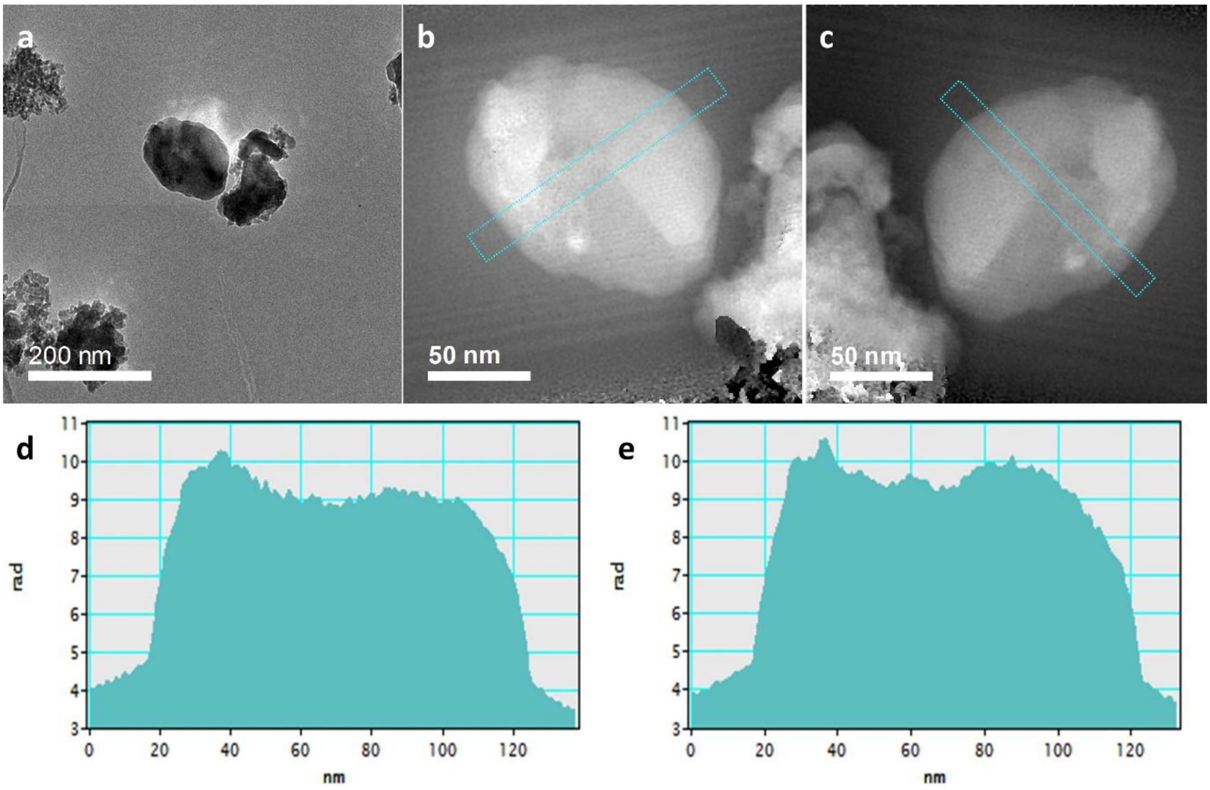

Figure S6 (iii) | a, Region of interest on TEM. **b,** Electron hologram of the particle unflipped, the field of view is 165 nm. **c,** Electron hologram flipped particle. **d and e,** Moreover a line profile in the 2 phase images reveal that as the amorphous carbon (aC) layer maintains its positive value (around 4-5 radians), for the non-magnetic particle we can't observe a change in the phase of the particle which stays at 10 radians both at flipped and unflipped phases.

Magnetic contours amplification

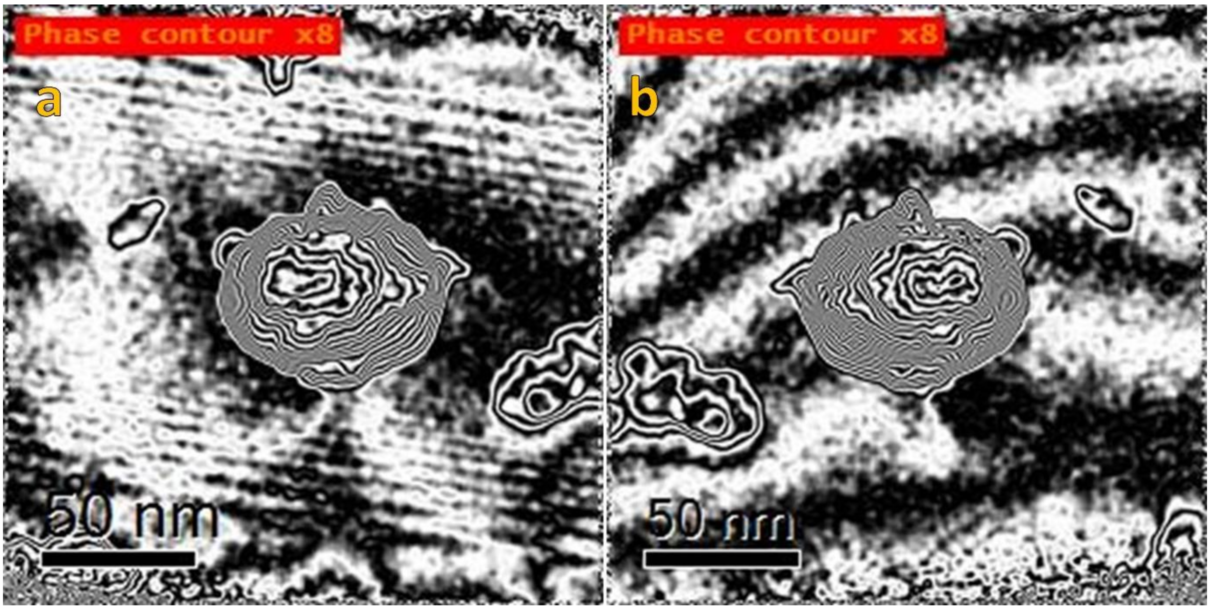

Figure S6 (iv) | Magnetic contours envelopes as seen on amplification of electron hologram phase to 8 times for **a,** un-flipped MENR 2**. b,** flipped MENR 2

Cross-section area calculation by Knud Thomsen's equation

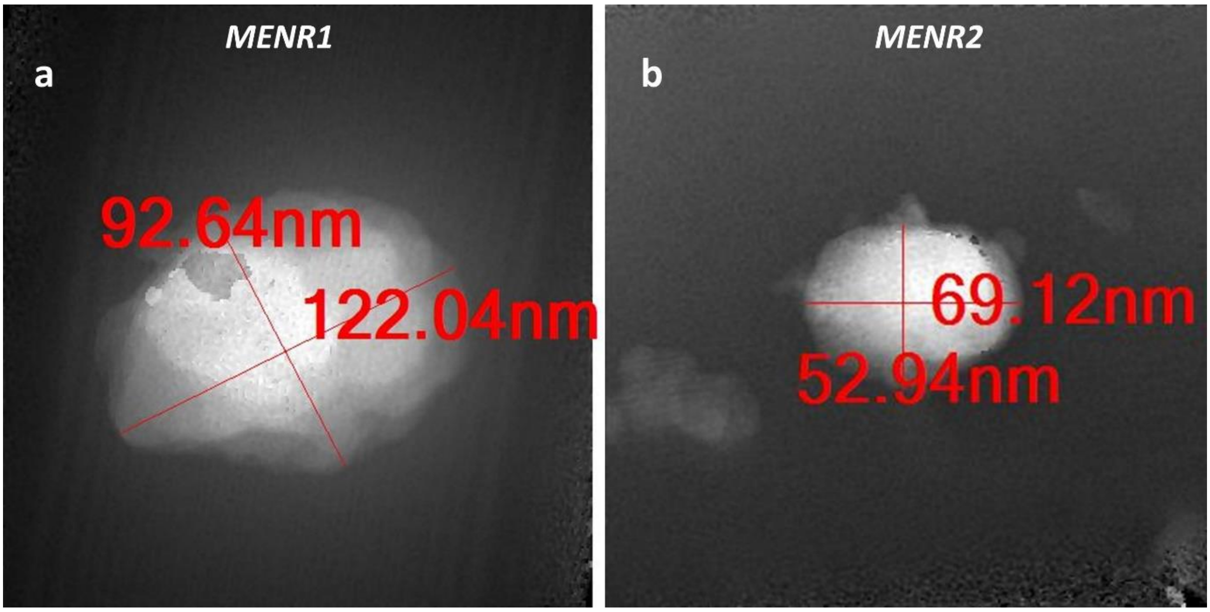

Figure S7 | Cross-section area calculation by Knud Thomsen's equation. MENRs dimention measurements from off-axis electron holography phase images **a,** MENR1 and **b,** MENR2 shapes corresponds to prolate spheroids.

Assuming the shape of both of the MENRs as prolate spheroids, according to Knud's Thomson equation, the surface area of the ellipsoids can be calculated from the equation below [34]:

Surface area = $\frac{4\pi*(a^p*b^p+a^p*c^p+b^p*c^p)^{1/p}}{3}$

Where $p \approx 1.6075$ yields a relative error of at most 1.061% (Knud Thomsen's formula)

For Fig. S7 (A) i.e. MENR1,

a = $\frac{122.04}{2}$ nm = 61.02 nm

b = $\frac{92.64}{2}$ = 46.32 nm

c = b = 46.32 nm

Surface area calculated for MENR1 = 3.28 X 10^{-14}Sq.m

For Fig. S7 (B) i.e. MENR2,

a = $\frac{69.12}{2}$ nm = 34.56 nm

b = $\frac{52.94}{2}$ = 26.47 nm

c = b = 26.47 nm

Surface area calculated for MENR2 = 1.06 X 10^{-14}Sq.m

Ferromagnetic hysteresis Curve of bulk sample

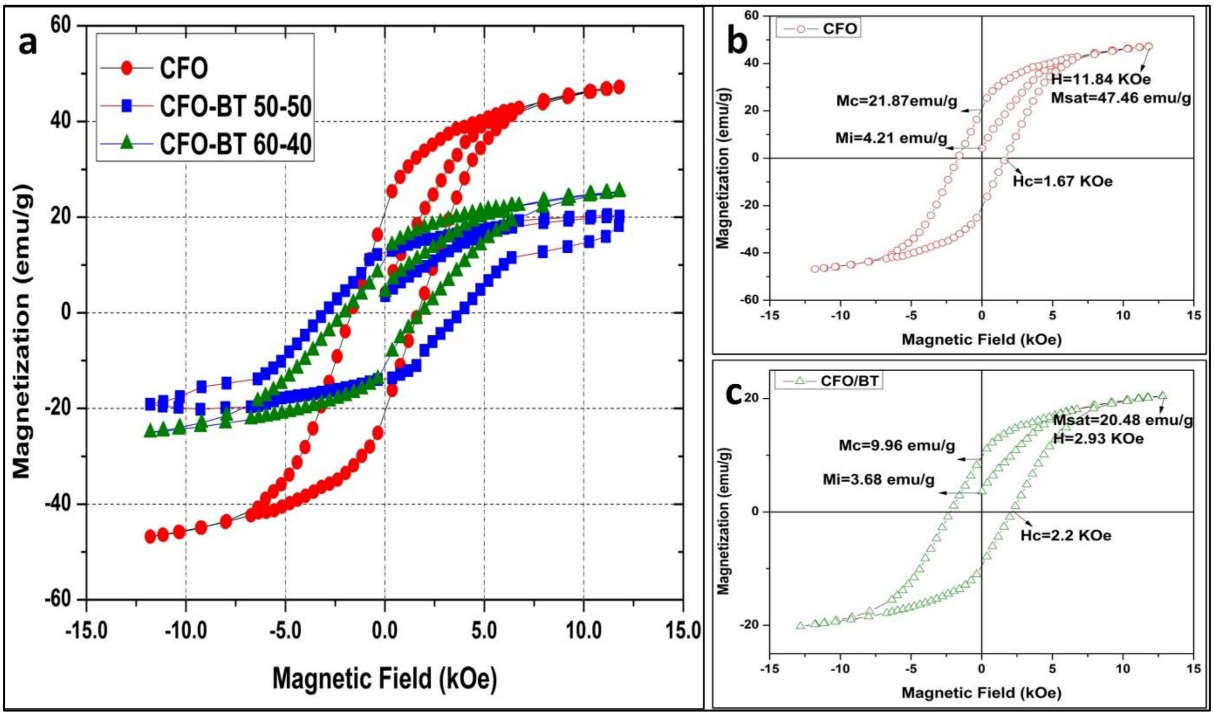

Figure S8 | Ferromagnetic hysteresis Curve [26] **– a,** The hysteresis measurement results show the ferromagnetic behaviour of bulk cobalt ferrite nanoparticles with peak magnetization of 51emu/g whereas after coating with different amount of barium titanate the peak magnetization decreases to 22emu/g with 60% CFO-40%BT and 18.4emu/g with 50%CFO-50%BT. **b and c**. Hysteresis measurements of cobalt ferrite nanoparticles and MENR respectively.

Electron holography at low magnification for low magnetic induction (50 Oe) due to objective lens off condition

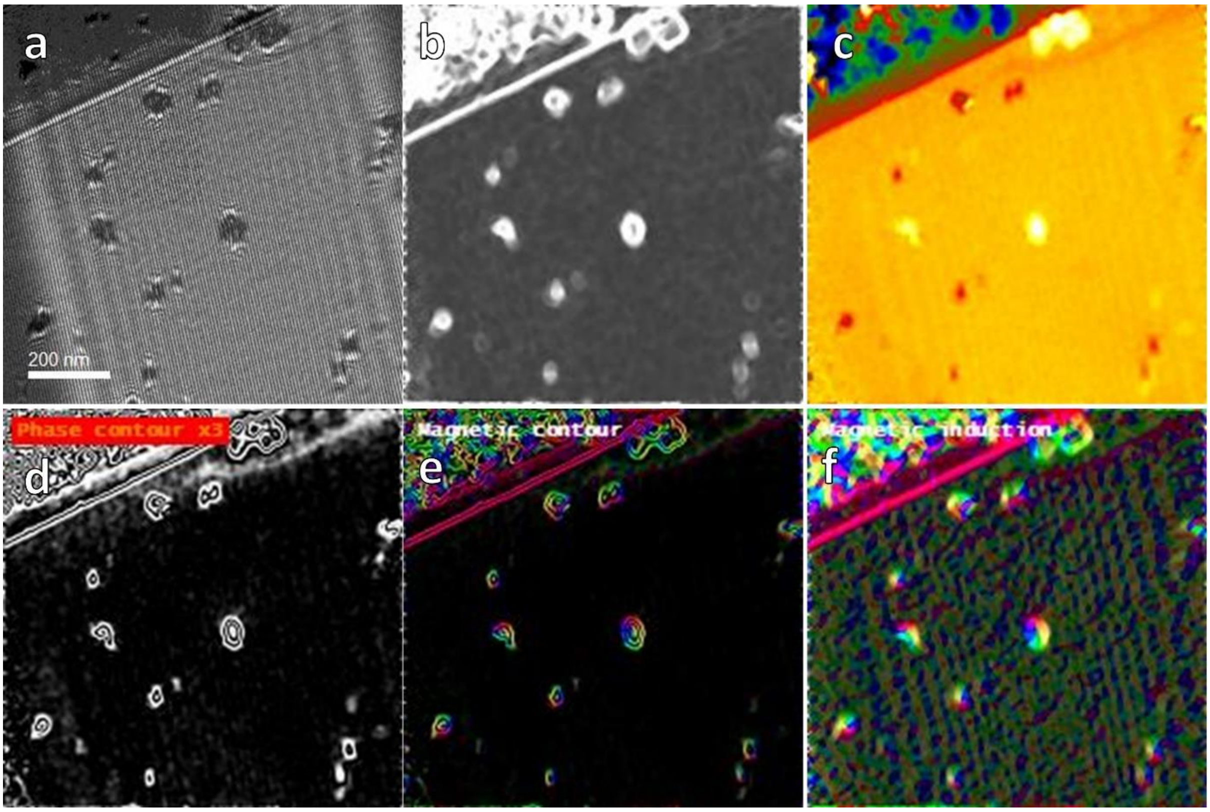

Figure S9 | Electron holography at low magnification for low magnetic induction (50 Oe) due to objective lens off condition: a, Single core-shell nanoparticle being analyzed under Fresnel fringes of 1µm. **b and c,** Extracted electromagnetic phase uncoloured and coloured. **d,** Phase contours magnified 3 times. **e,** Magnetic contours and **f,** Magnetic induction. The main limitation at this magnification level is the lack of visibility of distinction in core and shell of the MENR. Difference in core and shell electromagnetic contribution cannot be analysed at this magnification.

Cytotoxicity test Data with Human epithelial cell and NG108 rat neuronal cells

Cytotoxic test with Human Epithelial cells (HEP2)

Procedure details

1. MTS assay was performed for cytotoxicity test.

2. Human Epithelial cell line (Hep2) was used for the test.

3. Briefly, 10^5 cells were seeded in each well in 96 well plate with 100 µl of culture media. After 24 hour, media was replaced with media containing the samples in different concentration. The concentration used were 2 µg/ml, 10 µg/ml, 20 µg/ml, 50 µg/ml, 100 µg/ml, 200 µg/ml, 500 µg/ml and 1 mg/ml. The cells with samples were

incubated for 24 hour. 4. Then, the media was replaced with 100 µl of fresh media and 20 µl of MTS solution was added to each well. After incubating for 4 hour, absorbance at 490 nm was measured using Biotek Plate reader.

The MTS [3-(4,5-dimethylthiazol-2-yl)-5-(3-carboxymethoxyphenyl)-2-(4-sulfophenyl)-2H-tetrazolium] tetrazolium compound is bioreduced by metabolically active cells in to a coloured formazan product that is soluble in tissue culture medium. This conversion is accomplished by NADPH or NADH produced by dehydrogenase enzymes in metabolically active cells. The data is presented in Supplementary material- (Fig. S10) of this article. Cell viability test on time dependent d.c. and a.c. magnetic field application in presence of cells and CSMEN was also measured with concentration of 50 µg/ml. The cytotoxicity is always in control and hence depicts the safe usage of CSMEN for biomedical applications. Cell viability more than control i.e. the number of live cells counted after each experiment shows results higher than what needed for bio-compatibility confirmation.

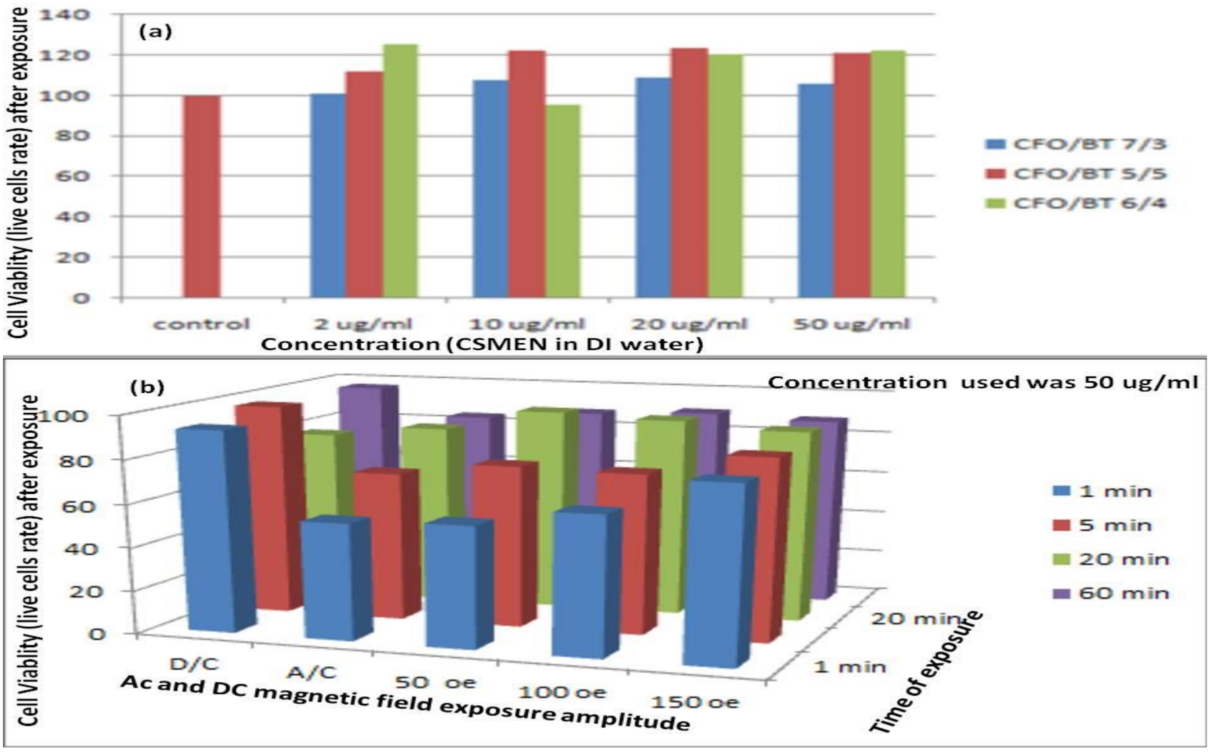

Figure S10 (i) | Cytotoxicity test Data [26]**: a,** MTS assay was performed for different composition of CSMEN, the graph shows the rate of live cells counted after the exposure of cells with CSMENs, **b,** Time and magnetic field intensity dependent Cytotoxicity test- The graph shows the rate of live cells counted after the exposure of cells with MENRs and exposed to AC & DC magnetic field with 50µg/ml concentration.

Cytotoxicity test with NG108 Rat Neuronal cells

Cytotoxic test with Rat neuronal cells (NG-108)

Procedure Details

Adenosine 5′-triphosphate (ATP) bioluminescent somatic cell assay kit. Sigma-Aldrich Cat.# FLASC-1KT.

ATP Assay reagent preparation

1. The CellTiter-Glo® Buffer and CellTiter-Glo® Substrate were thawed and equilibrate to room temperature prior to use.

2. 10ml (for Cat.# G7570) of CellTiter-Glo® Buffer was transferred into the amber bottle containing CellTiter-Glo® Substrate to reconstitute the lyophilized enzyme/substrate mixture. This forms the CellTiter-Glo®Reagent.

3. Mixing was done by gently vortexing to obtain a homogeneous solution. The CellTiter-Glo®Substrate.

ATP Assay Protocol

1. Microwell assay plates containing rat neuronal cells (NG -108) cells in culture medium were set up at desired density.

2. Test compounds and vehicle controls were added to appropriate wells so that the final volume is 100 μl in each well for 96-well plate.

3. 10^6 Cells were cultured for the desired test exposure period.

4. Plates were equilibrate to ambient temperature for 30 min to ensure uniform temperature across plate during luminescent assay.

5. CellTiter-Glo® Reagent were added in an equal volume (100 μl per well for 96-well plates) to all wells.

6. The content was mixed for 2 minutes on an orbital shaker to induce cell lysis.

7. The plate were incubated at room temperature for 10 minutes to stabilize luminescent signal and the luminescence was recorded.

The measurement of ATP using firefly luciferase is the most commonly applied method for estimating the number of viable cells. ATP has been widely accepted as a valid marker of viable cells. When cells lose membrane integrity, they lose the ability to synthesize ATP and endogenous ATPases rapidly deplete any remaining ATP from the cytoplasm. Although luciferase has been used to measure ATP for decades, recent advances in assay design have resulted in a single reagent addition homogeneous protocol that results in a luminescent signal that glows for hours.

The ATP detection reagent contains detergent to lyse the cells, ATPase inhibitors to stabilize the ATP that is released from the lysed cells, luciferin as a substrate, and the stable form of luciferase to catalyze the reaction that generates photons of light. Supplementary information (Fig. S10(ii) shows minimal decrease in ATP content of NG108 rat neuronal cells exposed to resazurin for 4 and 24 hours with concentration of MENR in aqueous medium as (2-16 µg/ml).

Luminescence count instrument description

Plate Type - 96 WELL PLATE, Read - Luminescence Endpoint (Temp 22.8 degree celcius), Full Plate: Integration Time: 0:01.00 (MM:SS.ss), Filter Set 1, Emission: Hole, Optics: Top, Gain: 135, Read Speed: Normal, Delay: 100 msec, Extended Dynamic Range, Read Height: 1 mm

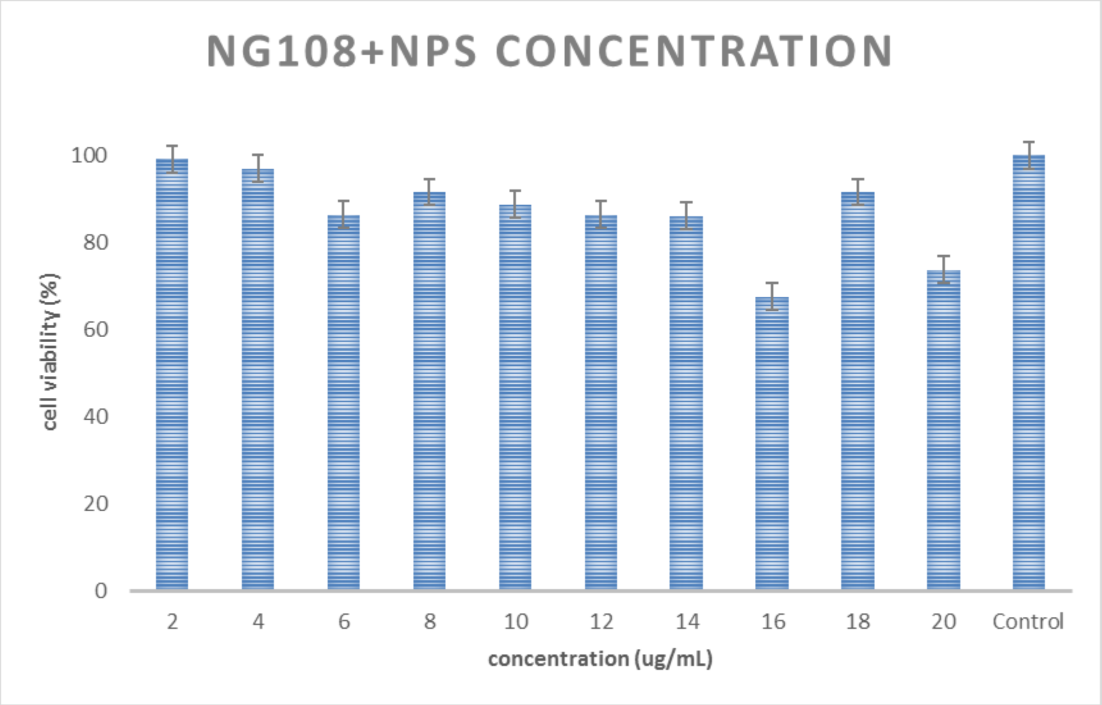

Figure S10 (ii) | NG108+MENR concentration study plate 1 (2-16 µg/ml): The data shows minimal decrease in ATP production, except the 16 and 20 µg/mL concentrations. Results can conclude that the cells continue to live in the presence of MENR for 24 hour.

UV-vis spectrophotometry results

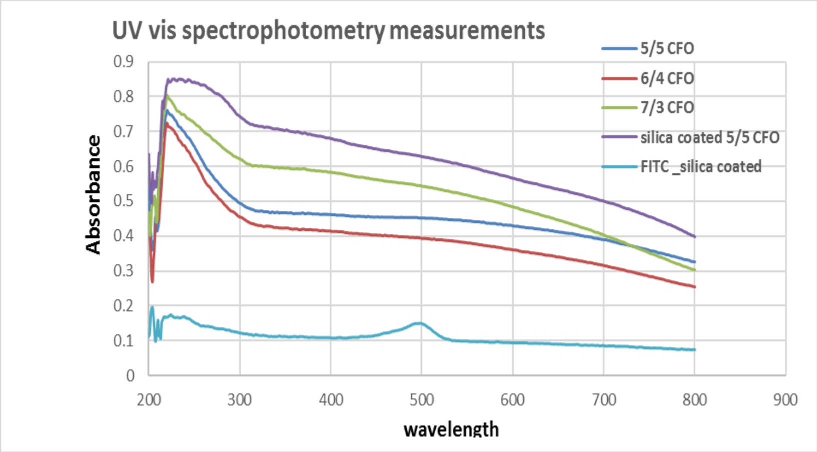

UV-vis spectrophotometry results for FITC coating confirmation on silica coated CSMEN since peak can be observed at 520 nm which is for green fluorescence emission of FITC

Supplementary Movies

Supplementary movie (Mov.1): The videos of the MENR-cellular targeted dynamic interactions have been submitted in the supplementary movie (Mov.1), when an a.c. magnetic field is applied, the MENR acts as a localized electric periodic pulse generator and can permeate (at 50 Oe, 60 Hz, above a threshold) a series of cells and align them to form an equipotential mono-array or cell patterning via inter-cellular signalling.

In supplementary movie (Mov.2), when under excitation of an a.c. magnetic field (40 Oe, 30 Hz), these nanorobots can be dynamically driven to an area or a targeted cell, avoiding other cells on the pathway, despite of cell density. They can be immobilized at any moment by switching off the magnetic field, which can be seen at the end of the video (Mov.2).

In supplementary movie (Mov.3), it can be seen that under dc magnetic field (-50 Oe) excitation, the MENRs act as thrust generator and induce motion in a group of cells and steer them to desired distances and will be stopped if magnetic field is switched off or intensity of magnetic field reduces. Also in supplementary movie (Mov.4), it can be seen that that MENRs can uniquely actuate suspended cells without disturbing any of the adhered cell area under dc magnetic field (-50 Oe) excitation.

References

33. Betal, S. et al. $BaTiO_3$ Coated $CoFe_2O_4$–Core-Shell Magnetoelectric Nanoparticles (CSMEN) Characterization. *Integrated Ferroelectrics***166**, 225 – 231 (2015)
34. Klamkin. M. S. Elementary Approximations to the Area of N-dimensional Ellipsoids. *American Mathematical Monthly*. **78**, 280 - 283 (1971).
